# P02.83. Mindfulness meditation in community dwelling older adults with postherpetic neuralgia

**DOI:** 10.1186/1472-6882-12-S1-P139

**Published:** 2012-06-12

**Authors:** R Meize-Grochowski, A Prasad, C Murray-Krezan, R Schrader, M DuVal, B Smith, C Herman

**Affiliations:** 1 College of Nursing, University of New Mexico, Albuquerque, USA; 2 Section of Integrative Medicine, School of Medicine, University of New Mexico, Albuquerque, USA; 3 Clinical & Translational Science Center, University of New Mexico, Albuquerque, USA; 4 Center for Life, University of New Mexico, Albuquerque, USA; 5 Psychology Department, University of New Mexico, Albuquerque, USA; 6 Division of Geriatrics, School of Medicine, University of New Mexico, Albuquerque, USA

## Purpose

This pilot study compared usual care alone with usual care plus meditation in relation to anxiety, depression, pain, and quality of life in community-dwelling older adults with postherpetic neuralgia (PHN). PHN may occur after shingles, and has been described as one of the most intractable neuropathic pain disorders. Older adults develop PHN more often than younger adults. Mindfulness meditation, found to be beneficial in the management of some chronic pain conditions, has not been specifically examined in older adults with PHN.

## Methods

Using a two-group pretest-posttest design with repeated measures, data were collected at entry to the study (Time 1), after a 2-week control period (Time 2), and 6 weeks later (Time 3). After Time 2 testing, participants were randomly assigned to usual care alone or usual care plus meditation. Daily pain diaries were kept by all participants for 8 weeks.

## Results

The 27 study participants were between 55 and 90 years of age, with a mean of 72 years. Fifteen participants were female, and 12 were male. Fourteen participants were White, 11 were Hispanic, and 2 were American Indian. At entry to the study, 18 participants reported moderate or greater pain seven days per week due to PHN. Data analysis included repeated measures ANOVA for the three time periods. Although no statistically significant differences were found for time or interaction effects, trends indicating a favorable response to mindfulness meditation over time were identified in the majority of the outcome variables.

## Conclusion

Participants were able to commit to the protocol for the duration of the study. The small sample size is a limitation; improvement in either group could be due to the natural course of PHN or even investigator attention. Future studies will include a sample size that is powered to detect significant differences at α=.05.

